# Ellagitannins (Ellagic Acid, Urolithin A, Urolithin B) Inhibit the Catalytic Activity of Human Recombinant Metalloproteinase 9

**DOI:** 10.5812/ijpr-148332

**Published:** 2025-03-09

**Authors:** Nigar Houssein-Zadeh, Leila Sadeghi, Gholamreza Dehghan

**Affiliations:** 1 Department of Biology, Faculty of Natural Sciences, University of Tabriz, Tabriz, Iran

**Keywords:** Recombinant Human Matrix Metalloproteinase-9, Ellagic Acid (EA), Urolithin A (Uro A), Urolithin B (Uro B), Mixed Inhibition

## Abstract

**Background:**

Ellagitannins are well-recognized for their antioxidant, chemopreventive, anti-inflammatory, and neuroprotective efficacy. Due to their poor absorption and extensive catabolism, it is proposed that urolithins, as ellagic acid (EA) metabolites, are the real active molecules exerting these biological functions.

**Objectives:**

This research evaluated the inhibitory effects of EA, urolithin A (Uro A), and urolithin B (Uro B) on the activity of recombinant human matrix metalloproteinase 9 (rhMMP-9). Dysregulation of MMP-9 activity is directly involved in various pathologies; therefore, inhibition of this enzyme has clinical importance.

**Methods:**

The rhMMP-9 activity was measured by a standard protease assay with casein as the substrate in the presence and absence of natural compounds, and the corresponding kinetic parameters were calculated. Interaction affinity between the enzyme and each of the ellagitannins studied was determined by the surface plasmon resonance (SPR) method. Molecular docking was performed using the C-terminally truncated human pro-MMP-9 structure as the receptor protein (PDB ID 1L6J) to predict ligand-receptor interaction and visualize the in vitro results.

**Results:**

The rhMMP-9 assay showed that EA, Uro A, and Uro B demonstrated inhibitory activity with IC_50_ values of 17.14 µM, 33.29 µM, and 13.17 µM, respectively. Kinetic interaction parameters calculated using SPR analysis showed the lowest KD for Uro B (4.3 × 10^-5^ M), compatible with its IC_50_. KD values calculated were 11.3 × 10^-5^ M for EA and 6.7 × 10^-5^ M for Uro A. A mixed type of inhibition with a non-competitive-uncompetitive pattern for Uro A and Uro B and a competitive-non-competitive pattern for EA was revealed.

**Conclusions:**

Our results showed the promising inhibitory potential of EA, Uro B, and Uro A to affect the catalytic activity of the MMP-9 enzyme and also confirmed the fibronectin domain as a potential site for drug design against MMP-9.

## 1. Background

Matrix metalloproteinase 9 (MMP-9) belongs to a family of Zn-dependent endopeptidases and is involved in various biological processes due to its specific capacity to degrade the extracellular matrix (ECM) (1, 2). The catalytic activity of this enzyme causes degradation of the ECM, which acts as a physical barrier separating cells, and facilitates tumor cells in penetrating the basement membrane and entering the blood and lymphatic vessels. Therefore, MMP-9 activation is a primary mechanism by which cancerous cells begin to grow in other parts of the body, a process known as metastasis (3).

Given the important role of MMP-9 in cancer pathologies, the development of its inhibitors and targeting of MMP-9 is a current priority. Most studies have evaluated the expression of MMP-9 protein in cell lines and tissue lysates without specific assays of the protease enzyme (4, 5). To our knowledge, none of the MMP-9 inhibitors have been finally approved for use as a drug with minor side effects (6). Many researchers are working on the development of natural inhibitors of MMP-9 (3, 7). Some compounds isolated from natural herbal sources, such as flavonoids, alkaloids, phenolic compounds, glycosides, and polyphenols, can inhibit MMP-9 at the gene expression level. Ellagitannins and their derivatives, such as ellagic acid (EA), urolithin A (Uro A), and urolithin B (Uro B), are among these bioactive phytochemicals.

Ellagic acid is reported as a chemopreventive agent with anticancer effects (8, 9) through mechanisms such as inhibiting cancer cell proliferation, promoting apoptosis, exhibiting anti-inflammatory activity, and preventing DNA damage from oxidative stress and carcinogenic agents (10). Several studies have shown the anti-inflammatory, neuroprotective, and anticancer effects of EA by reducing MMP-9 expression (11, 12).

Orally administered ellagitannins are less digestible, and unabsorbed ones are transformed into urolithins in the large intestine (13). Due to their low bioavailability, it is proposed that urolithins, as a product of their catabolism, may be responsible for the actual biological action in the body (14). The influences of urolithins on the expression or activity of MMP-9 are not yet well understood. However, published results have confirmed that Uro A, Uro B, and Uro C can reduce MMP-9 expression in neutrophils in vitro (15), and Uro A and Uro B decreased MMP-9 expression in endometriotic cell cultures (16). A significant decrease in MMP-9 activity treated with Uro A was demonstrated in a colorectal cancer cell line previously (17). Urolithins also attenuate inflammation induced by hemozoin and TNF-α (18).

It can be concluded that EA and its derivatives reduce MMP-9 expression by affecting transcription factors and subsequent processes.

## 2. Objectives

Based on the effects of ellagitannins on the reduction of MMP-9 expression, this research aimed to examine the specific effects of EA and its derivatives, Uro A and Uro B, on the catalytic activity of recombinant human MMP-9 (rhMMP-9) using the standard protease activity assay. Kinetic parameters of rhMMP-9 protease activity were estimated in the presence and absence of the ellagitannins. Surface plasmon resonance (SPR) measurements and a molecular docking study were also conducted to understand the possible interactions between these natural molecules and the MMP-9 enzyme, providing new structural insights for drug design.

## 3. Methods

### 3.1. Materials

All chemicals used were of analytical grade and were provided by Merck (Germany). Isopropyl-β-D-thiogalactopyranoside (IPTG) was obtained from Sigma-Aldrich (USA). Ellagic acid, Uro A, and Uro B were prepared in the laboratory of Dr. Iranshahi at Mashhad University of Medical Sciences. Ellagitannin powders were first dissolved in DMSO to achieve a concentration of 1 mg/mL and further diluted with phosphate buffer to prepare working dilutions for use in experiments.

### 3.2. Expression and Purification of Protein

**Figure 1.**
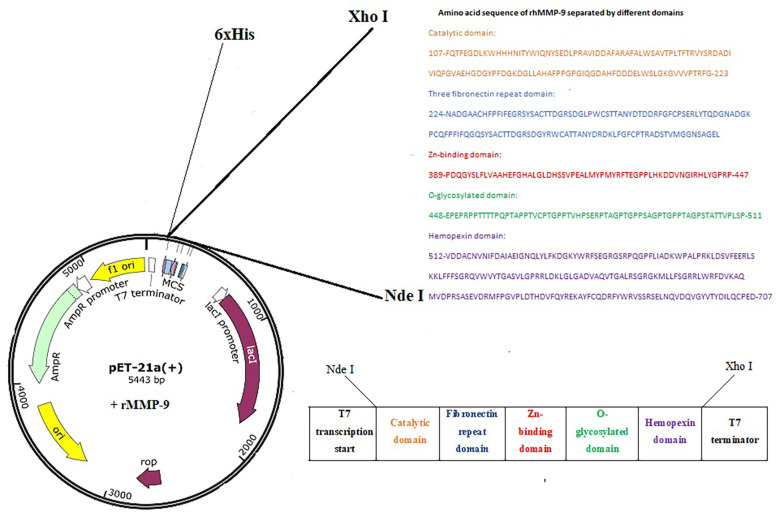
Plasmid system encoding full-length recombinant human matrix metalloproteinase 9 (rhMMP-9) and amino acid sequence of rhMMP-9 divided by domains

*Escherichia coli* BL21 (DE3) cells containing the plasmid vector pet21a (+)-rhMMP-9 were used in this study. The cloned and expressed rhMMP-9, containing amino acid residues from 107 to 707, consisted of the catalytic domain (Phe107 to Gly223), a three fibronectin repeat domain (Asn224 to Cys388), a Zn^2+^-binding domain (Pro389 to Pro447), an O-glycosylated domain (Glu448 to Pro511), and a hemopexin domain (Val512 to Asp707) (Figure 1). The recombinant bacteria were used for the expression of the rhMMP-9 gene according to previously optimized conditions (19).

A single colony of this microorganism was cultured in 10 mL of Luria-Bertani (LB) medium containing 100 µg/mL of ampicillin and incubated for 16 hours at 37°C with shaking to prepare a preculture. One milliliter of preculture was cultured into 100 mL of ampicillin-containing LB medium and incubated in a shaker incubator at 37°C until the optical density reached 0.5 - 0.6 at 600 nm. The MMP-9 expression was induced by adding 0.1 mM IPTG to the culture media and incubating for 4 hours. Cells were collected by centrifugation (2500 g, 5 min), lysed with 20 mM Tris buffer (pH 7), and sonicated twice (10 min, 4°C) with a 10-minute rest interval at 4°C. Sonication was performed using a professional ultrasonic cleaner Sonic 3MX by James Products Europe (160 W). Cells were centrifuged again (12000 g, 10 min, 4°C). The supernatant fraction containing the desired protein was purified by loading onto a Ni-NTA column. The Ni-NTA column was equilibrated with lysis buffer (pH 7). A linear gradient of washing buffer (pH 8.0) containing 50 mM Tris, 300 mM NaCl, and imidazole (30, 40, and 50 mM) was used to wash the column. rhMMP-9 protein was eluted with the same buffer containing 60 mM imidazole. Enzyme fractions were collected and dialyzed twice with 20 mM Tris buffer (pH 7) (20).

The rhMMP-9 expression and purification were validated by SDS-PAGE (12.5% polyacrylamide). The sample was diluted with 20 mM Tris (pH 7) buffer and 5x sample buffer. The mixture was maintained for 5 minutes in boiling water, and 15 µL of this solution was transferred into the lane. Gel staining was accomplished with Coomassie Brilliant Blue R-250 reagent (21).

### 3.3. Protease Activity Assay

To determine the hydrolyzing activity of rhMMP-9, casein was used as a substrate. A casein solution (1% w/v) was added to the extracted and purified rhMMP-9, and the final volume was adjusted to 1 mL with Tris buffer (pH 7). The expression product and substrate mix were incubated with shaking for 5 minutes at 37°C. To stop the process, 1 mL of cold trichloroacetic acid (TCA, 15%) was added to the mixture. This mixture was then kept at -20°C for 10 minutes and centrifuged twice (8000 g, 5 min, 4°C) to separate soluble peptides. The pellet was discarded, and the absorbance of the supernatant soluble peptides fraction was evaluated at 280 nm. All spectrophotometric measurements were accompanied by a blank assay in the absence of the enzyme. Enzyme activity was calculated from the tyrosine standard curve and expressed as mean ± SD in units of [µmol/min] after at least five repeats (22). SPSS software version 9 was used for data interpretation, and all graphs were drawn and kinetic parameters calculated using an Excel spreadsheet software program. The Quest Graph IC_50_ Calculator was used for the calculation of IC_50_ values.

### 3.4. Protein Concentration Measurement

Measurement of rhMMP-9 concentration was done using a routine Bradford test procedure (23). Several albumin standard solutions were prepared, and their absorbance at 595 nm was read after their reaction with Bradford reagent. The absorbance of the expression product of rhMMP-9 (supernatant fraction obtained in the process of protein expression before and after purification) after its incubation with Bradford reagent was also measured, and its concentration was obtained from the standard Bradford curve. The culture medium inoculated with recombinant *E. coli* BL21 (DE3) without stimulation of protein expression by IPTG was used as a control in all of the experiments.

### 3.5. Surface Plasmon Resonance Measurements

A dual-flow channel MP-SPR Navi 210A analyzer (BioNavis Ltd., Tampere-region, Finland) was used to evaluate SPR kinetic parameters on gold chips (BioNavis Ltd., Finland). A solution of both EDC/NHS ((N-(3-dimethylaminopropyl)-N′-ethylcarbodiimide hydrochloride) (0.2 M) and NHS (N-hydroxysuccinimide) (0.05 M)) was employed for the activation of rMMP-9 enzyme carboxylic groups. Immobilization of the rMMP-9 enzyme on the CMD Au chip was accomplished by a standard amine coupling protocol. For blocking unreacted sites, ethanolamine–HCl (1 M, pH 8.5) reagent was used. Five different concentrations of Uro A, Uro B, and EA, including 5, 10, 20, 30, and 40 µM for each, were injected at a flow rate of 30 µL/min for 300 seconds (24).

### 3.6. Molecular Docking

The protein structure of human matrix metalloproteinase MMP-9 (gelatinase B) with PDB ID 1L6J was downloaded from the RSCB PDB database (25). The protein molecule was protonated, and water molecules were removed. Ligand structures were obtained from the PubChem site with code numbers 5281855 for EA, 5488186 for Uro A, and 5380406 for Uro B. Ligand structures were optimized using Gaussian View 03 software. Docking was performed with AutoDock 4.2 software. The blind dock was accomplished with a grid box having the number of grid points = 126 × 126 × 126 and with grid point spacing = 0.375 Å. Visualization of docking poses was done using Discovery Studio 2021 and Chimera UCSF.

## 4. Results

Medicinal herbs and derived natural compounds may have practical value as substitute therapeutic agents. Several natural products have been applied as effective inhibitors for MMP-9 due to their specific activity and low toxicity. Empirical evidence shows that coumarins are important leading compounds for the development of anti-inflammatory and anti-cancer drugs (26). Since the MMP-9 enzyme is a central target in the treatment of inflammatory diseases, various types of cancers, and neurological disorders, finding appropriate inhibitors can aid in combating these diseases.

In this study, valuable data were gained concerning the structural and functional changes of rhMMP-9 as a result of interaction with ellagitannins; this could provide a novel outlook on the design of natural medicines based on inhibition mechanisms and offer additional insight into substituting synthetic chemotherapy compounds with herbal medicines.

### 4.1. Expression and Purification of Recombinant Human Matrix Metalloproteinase 9 from Escherichia coli

**Figure 2.**
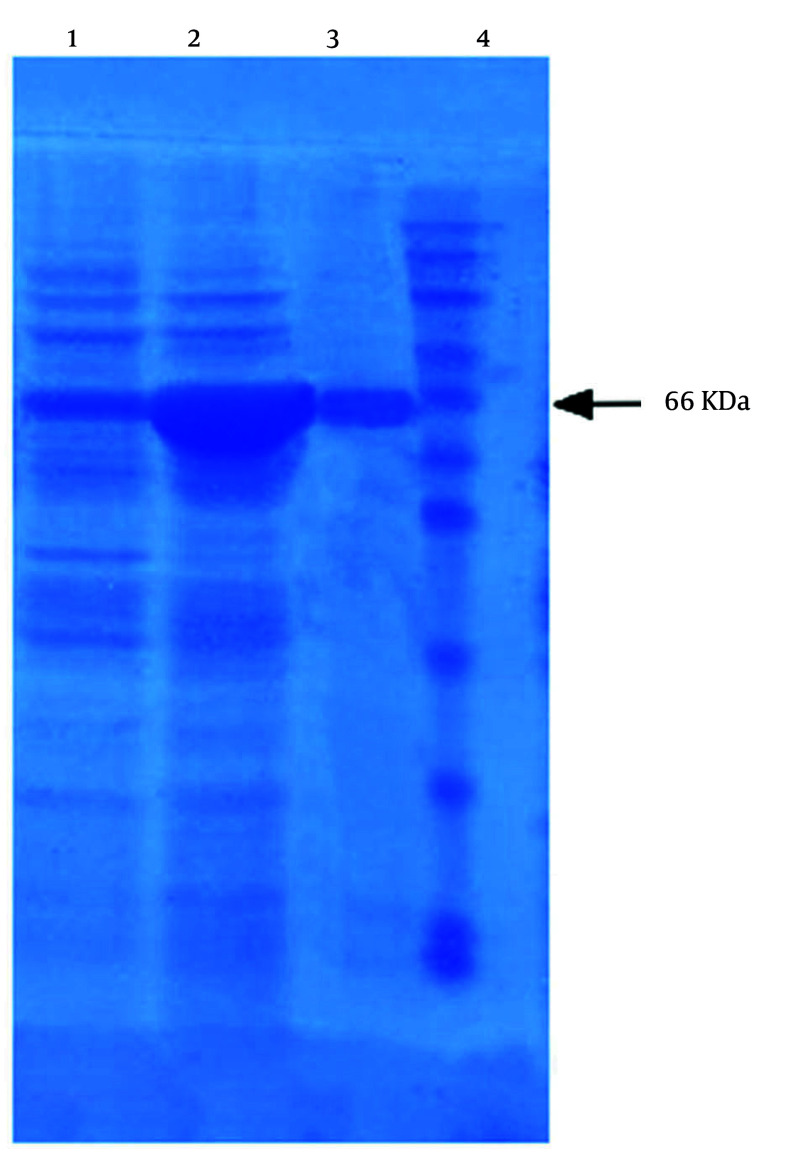
SDS-PAGE analysis of recombinant matrix metalloproteinase 9 (rMMP-9) expression product: Lane 1, before isopropyl-β-D-thiogalactopyranoside (IPTG) induction (2.3 mg/mL); lane 2, after IPTG induction (8.45 mg/mL); lane 3, pure protein [matrix metalloproteinase 9 (MMP-9); lane 4, molecular marker

Active full-length rhMMP-9 was successfully expressed by *E. coli* BL21 (DE3) cells through the expression vector pET21a-MMP-9 (Figure 1). Expression of rhMMP-9 in the soluble fraction was revealed by SDS-PAGE (Figure 2) and through monitoring catalytic activity. The protein content of the supernatant after expression was compared with an equal sample that did not receive IPTG during expression. Protein concentration was measured by the Bradford method, and results confirmed that more than 40% of the total proteome of bacteria is rhMMP-9 protein. The specific activity of the recombinant enzyme was calculated as the enzyme's activity per milligram of total protein and constituted 1.86 ± 0.03 µmol min^-1^ mg^-1^. Purification with a Ni-Agarose column was done according to the His-tag of the recombinant protein, and the purified protein was used for kinetic experiments.

### 4.2. Inhibition of Recombinant Human Matrix Metalloproteinase 9 Activity by Ellagic Acid and Its Derivatives

**Figure 3.**
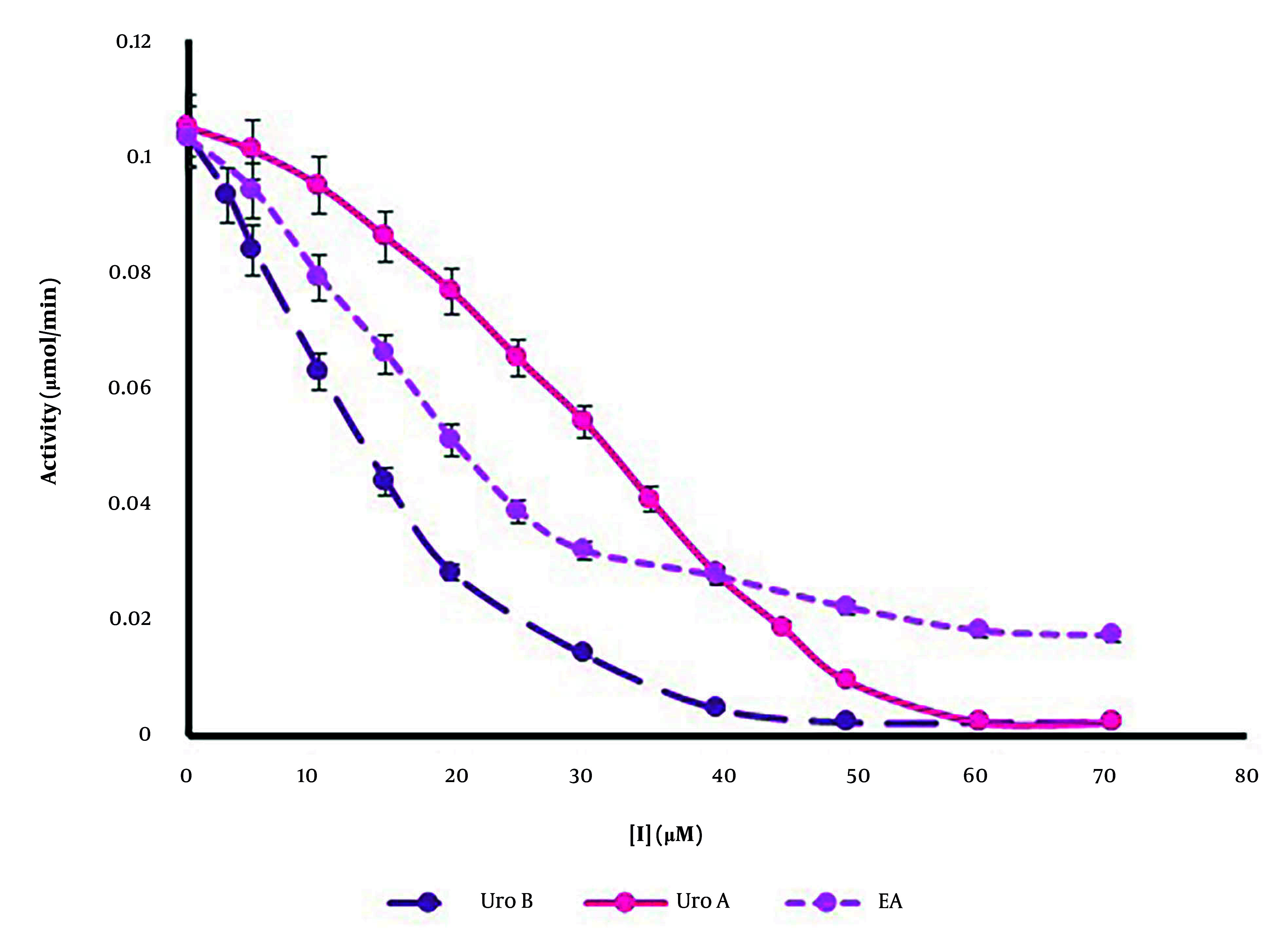
Dose-response curves of recombinant human matrix metalloproteinase 9 (rhMMP-9) activity inhibition at the presence of various concentrations of ellagic acid (EA), urolithin A (Uro A); and urolithin B (Uro B). Data were stated as mean ± SD (n = 6).

A cell-free casein degradation-based assay was employed to evaluate the influence of ellagitannins on the catalytic activity of the rhMMP-9 enzyme. By adding various fixed quantities of casein as a substrate and a fixed concentration of enzyme, the enzyme activity was determined in the presence of different concentrations of ellagitannins (ranging from 0 to 70 µM). The rate of tyrosine production by the protease activity of rhMMP-9 was assessed based on a calibration curve over 1 minute, which refers to enzyme activity. As shown in Figure 3, ellagitannins could inhibit rhMMP-9 activity in a concentration-dependent manner. The concentrations of the inhibitor that could inhibit 50% of the enzyme’s activity, or its IC_50_ values, were calculated as 13.17 µM, 33.29 µM, and 17.14 µM for Uro B, Uro A, and EA, respectively. Urolithin B showed the highest inhibition of rhMMP-9 based on protease activity measurements (Figure 3). Our results also demonstrated that Uro B and Uro A could entirely inhibit the catalytic activity of the enzyme at high concentrations, but the enzymatic function of rhMMP-9 does not reach zero in the presence of EA, which could reduce 90% of catalytic activity at a high concentration (60 µM).

### 4.3. Mixed Inhibition Mechanism of rhMMP-9 Activity by Ellagitannins

**Figure 4.**
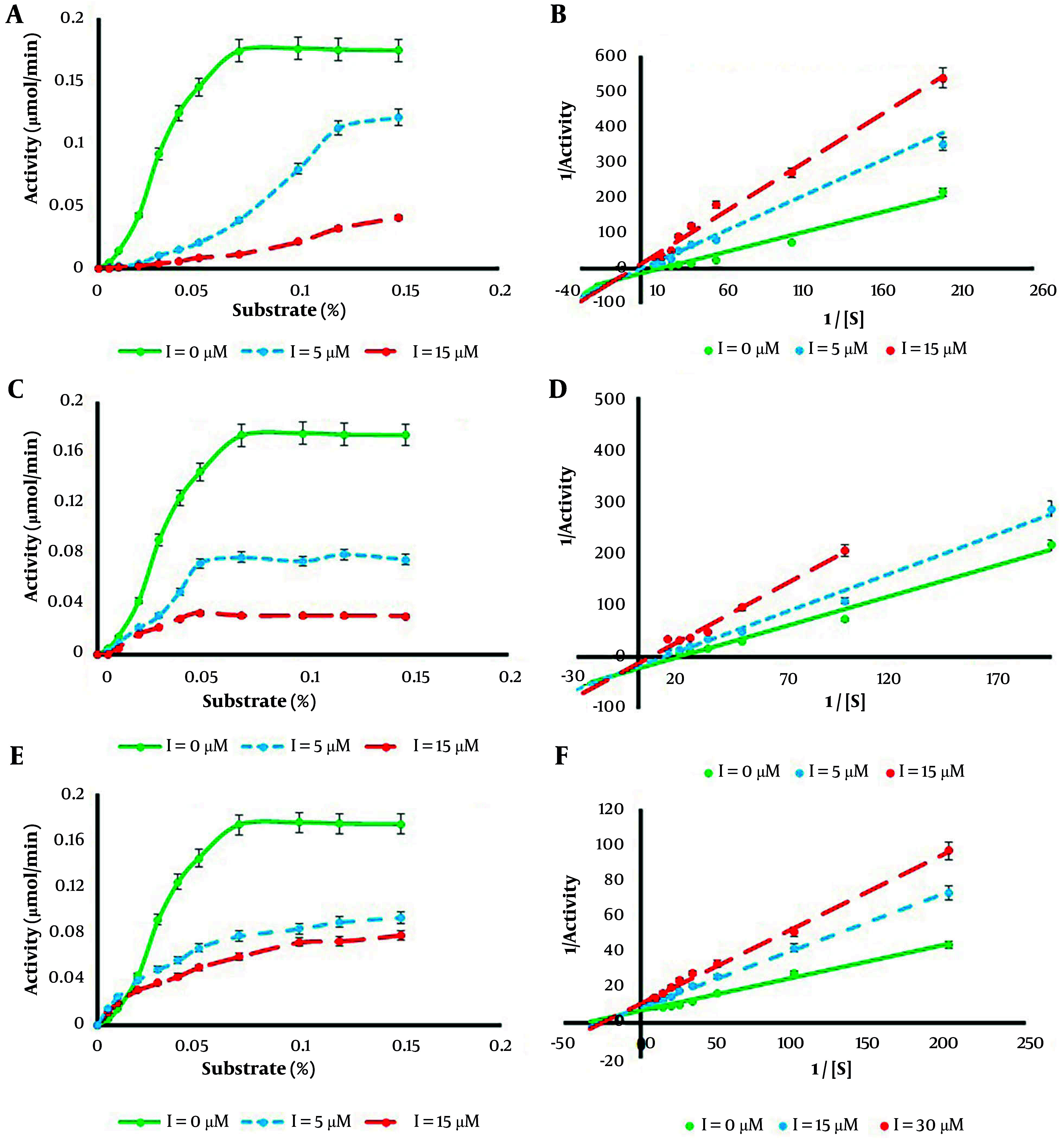
Michaelis-Menten curve [A for urolithin A (Uro A); C for urolithin B (Uro B); E for ellagic acid (EA)] and Lineweaver-Burk plot (B for Uro A; D for Uro B; F for EA) for recombinant human matrix metalloproteinase 9 (rhMMP-9) in the presence of various concentrations of ellagitannins. Values were expressed as mean ± SD (n = 5).

Measurement of rhMMP-9 enzyme casein degradation ability at different concentrations of ellagitannins demonstrated classic Michaelis-Menten saturation kinetics. The Michaelis-Menten expression describes the behavior of many enzymes at different concentrations of substrates. To compare the behavior of the enzyme in the presence and absence of inhibitors (ellagitannins), the Lineweaver-Burk equation or double reciprocal plot was used in this research. With the increase of substrate amount, as shown in Figure 4, an escalation in enzyme activity was observed; nevertheless, a notable decrease in enzyme activity at higher substrate concentrations was detected.

To examine the effects of inhibitors on the kinetic parameters of rhMMP-9, Michaelis-Menten saturation curves and Lineweaver-Burk plots were drawn in the presence of various concentrations of inhibitors (5 - 30 µM) and substrate range (0 - 0.3%) (Figure 4). It was shown that both the maximum velocity of the system and K_m_ decreased with increasing the concentration of Uro B from 5 to 15 µM. For Uro A, with the increase of inhibitor concentration from 5 to 15 µM, the maximum velocity of the system also decreased, but K_m_ increased. The EA also caused a reduction in V_max_ value, but the K_m_ constant decreased at the lower concentration of inhibitor (15 µM) and then remained constant at the higher concentration of inhibitor (30 µM). Table 1 summarizes the kinetic parameters for the rhMMP-9 enzyme in the presence and absence of inhibitors at different concentrations.

**Table 1.** System Parameters (V_max_ and K_m_) and System Factors (Ki, KI and α) at the Presence of Various Concentrations of Ellagitannins ^a^

System Parameters and Factors	Uro A (µM)			Uro B (µM)			EA (µM)		
	0	5	15	0	5	15	0	15	30
**V**_**max**_ **(µmol/min)**	0.175 ± 0.009	0.120 ± 0.006	0.04 ± 0.002	0.175 ± 0.009	0.079 ± 0.004	0.033 ± 0.002	0.175 ± 0.009	0.093 ± 0.005	0.078 ± 0.004
**K**_**m**_ ** (%)**	0.036 ± 0.002	0.085 ± 0.004	0.1 ± 0.005	0.036 ± 0.002	0.030 ± 0.002	0.020 ± 0.001	0.036 ± 0.002	0.03 ± 0.002	0.03 ± 0.002
**Ki**	12			22.5			24.85		
**αKi (KI)**	3.03			3.02			27.57		
**α**	0.25			0.13			1.11		

Abbreviations: Uro A, urolithin A; Uro B, urolithin B; EA, ellagic acid.

^a^ Each concentration was repeated 5 times and all data are represented as mean ± SD.

Comparison of the results and Lineweaver-Burk plots for rhMMP-9 in the presence of various concentrations of Uro A (Figure 4B), Uro B (Figure 4D), and EA (Figure 4F) demonstrated a mixed type of inhibition with different inhibitor constants. It is known that inhibitors can attach to the enzyme by two processes Equations 1 and 2.


E+I↔EI (Inhibitor constant Ki)

ES+I↔ESI [Inhibitor constant α Ki (KI)]

Therefore, Equations 3 and 4:


Ki=[E][I]/[EI]

αKi=KI=[ES][I]/[ESI]

Ki, or the dissociation constant for the EI complex, indicates how tightly the inhibitor is binding to the free enzyme [E] (Equation 3), while αKi (KI) represents the dissociation constant for the ESI complex, referring to how tightly the inhibitor is binding to the [ES] complex (Equation 4). Hence, two types of inhibitor constants, Ki and αKi, can be evaluated in each inhibition case according to secondary plots.

**Figure 5.**
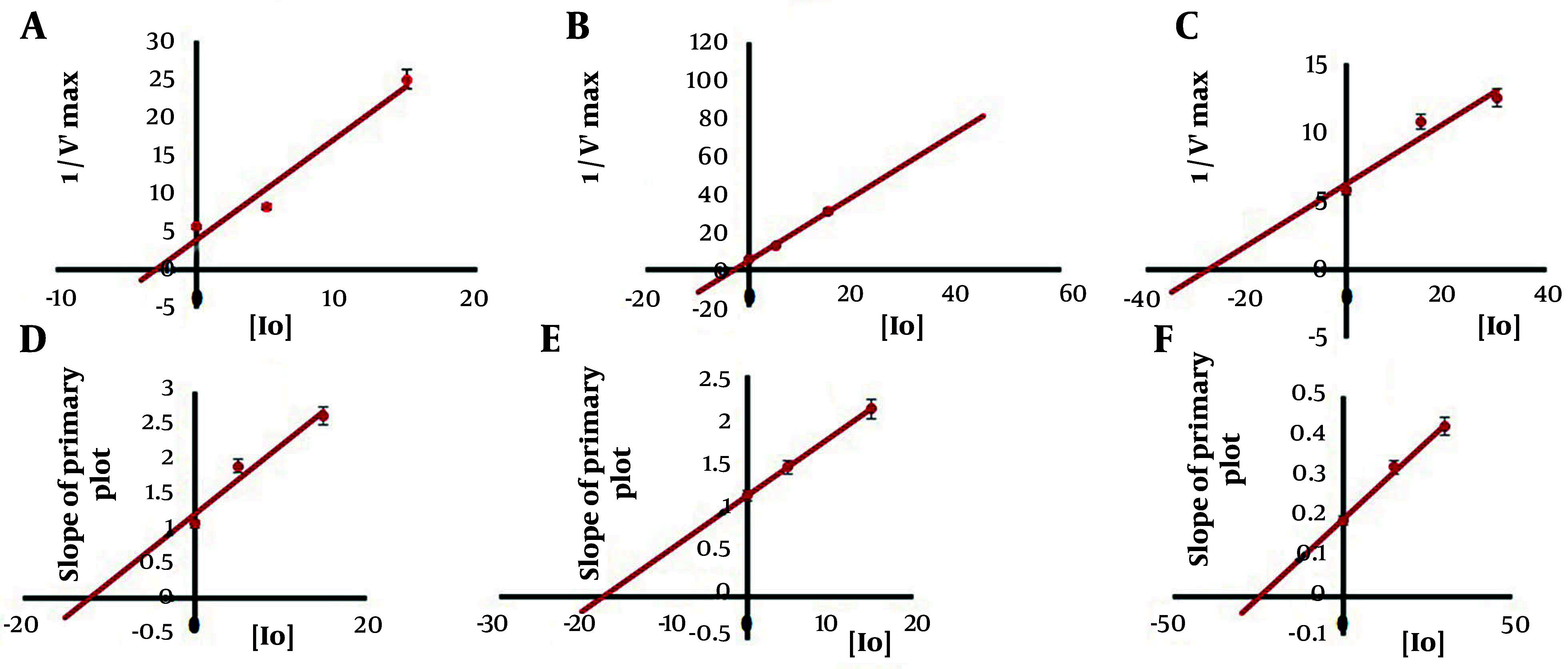
Secondary plots of 1/Vʹ_max_ against [Io] [A for urolithin A (Uro A), B for urolithin B (Uro B), C for ellagic acid (EA)] and secondary plots of the slope of the primary plot against [Io] (D for Uro A, E for Uro B, F for EA). Data were stated as mean ± SD (n = 5).

The first of these is a Dixon plot of 1/Vʹ_max_ against the concentration of inhibitor [I], from which the value of -αKi as the x-intercept can be determined (Figure 5A - C). The second is the slope of the primary plot (from the Lineweaver-Burk plot) against the concentration of inhibitor [I], from which the value of -Ki as the x-intercept can be determined (Figure 5D - F).

Factor of alfa (α) can be determined from Equation 5.


KI= α × Ki

Calculated values of Ki, KI, and α are given in Table 1 for each inhibitor. For all two urolithins tested KI < Ki and α is less than 1, so it could be concluded that these inhibitors demonstrated non-competitive-uncompetitive patterns of inhibition which is a specific type of mixed inhibition (27). This type of inhibition confirmed that urolithin molecules could attach to both free enzyme and enzyme-substrate complex but the inhibitor affinity to enzyme-substrate complex is more than to the free enzyme.

In the case of EA KI > Ki and α is more than 1, so it could be concluded that this inhibitor demonstrated competitive-non-competitive model of inhibition which is a specific type of mixed inhibition (27). This type of inhibition confirmed that EA could attach to both free enzyme and enzyme-substrate complex however the inhibitor affinity to the free enzyme is more than to enzyme-substrate complex.

### 4.4. Increased Affinity of Urolithin B to rhMMP-9 Based on SPR Results

**Figure 6.**
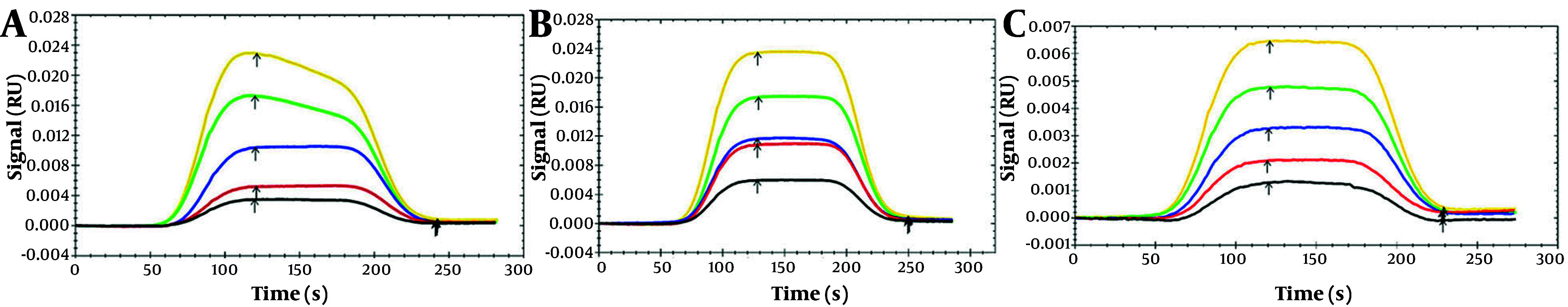
Sensograms of A, urolithin A (UA); B, urolithin B (UB) and C, ellagic acid (EA) binding to recombinant human matrix metalloproteinase 9 (rhMMP-9) at different concentrations at 37^°^C (5, 10, 20, 30, 40 µM)

The SPR technique, a current method for examining the interaction between ligand and protein, was employed in this research. As presented in Figure 6, association (Ka) and dissociation (Kd) constants were evaluated through sensorgrams (RU vs. time). Five identical concentrations for each of the three natural compounds were used (5, 10, 20, 30, 40 µM).

The equilibrium constant KD, which evaluates the affinity of a ligand to a protein, was determined using the formula: =Kd/Ka . A lower value of the KD constant indicates a higher interaction affinity between the protein and ligand. In this study, the KD values were measured for Uro A-rhMMP-9, Uro B-rhMMP-9, and EA-rhMMP-9 complexes to be 6.7 × 10^-5^ M, 4.3 × 10^-5^ M, and 11.3 × 10^-5^ M, respectively, at 37°C, as shown in Table 2. 

**Table 2.** KD values achieved by SPR; binding energies and binding positions of ellagitannins on MMP-9 obtained from molecular docking studies

Natural Molecules	KD (M)	Catalytic Domain		Fibronectin Domain	
		Binding Energy (kcal/mol)	Interaction Amino Acids	Binding Energy (kcal/mol)	Interaction Amino Acids
**Uro A**	6.7 × 10^-5^	-8.41	Leu 397, Val 398, His 401, Glu 402, Pro 415, Tyr 423, Arg 424	-7.44	Leu 212, Lys 214, Gly 215, Val 217, Ala 229, His 231, Cys 230, Phe 232, Thr 258
**Uro B**	4.3 × 10^-5^	-8.54	Leu 397, Val 398, His 401, Pro 415, Tyr 423, Arg 424	-6.72	Leu 212, Lys 214, Gly 215, Val 217, Ala 229, His 231
**EA**	11.3 × 10^-5^	-6.19	Met 422, Arg 424, Thr 426, Pro 429	-7.35	Glu 130, Val 217, Val 218, Pro 219, Pro 272, Tyr 277, Thr 331, Ala 333

Abbreviations: Uro A, urolithin A; Uro B, urolithin B; EA, ellagic acid.

The achieved results indicate that Uro A-rhMMP-9 and Uro B-rhMMP-9 complexes have lower KD constant values compared to the EA-rhMMP-9 complex, which means that urolithins, especially Uro B, have more affinity for the MMP-9 enzyme, while this affinity is noticeably reduced for the EA-rhMMP-9 complex.

### 4.5. Molecular Docking Analysis

To understand and predict the inhibition mechanism and identify the attachment sites of the three inhibitor molecules on rhMMP-9, we conducted molecular docking. The results showed that there are two potential binding positions for Uro A, Uro B, and EA molecules on the MMP-9 protein: The catalytic domain and the fibronectin domain, which exhibit the lowest binding energy compared to other binding positions. Table 2 summarizes the docking results for both binding positions.

**Figure 7.**
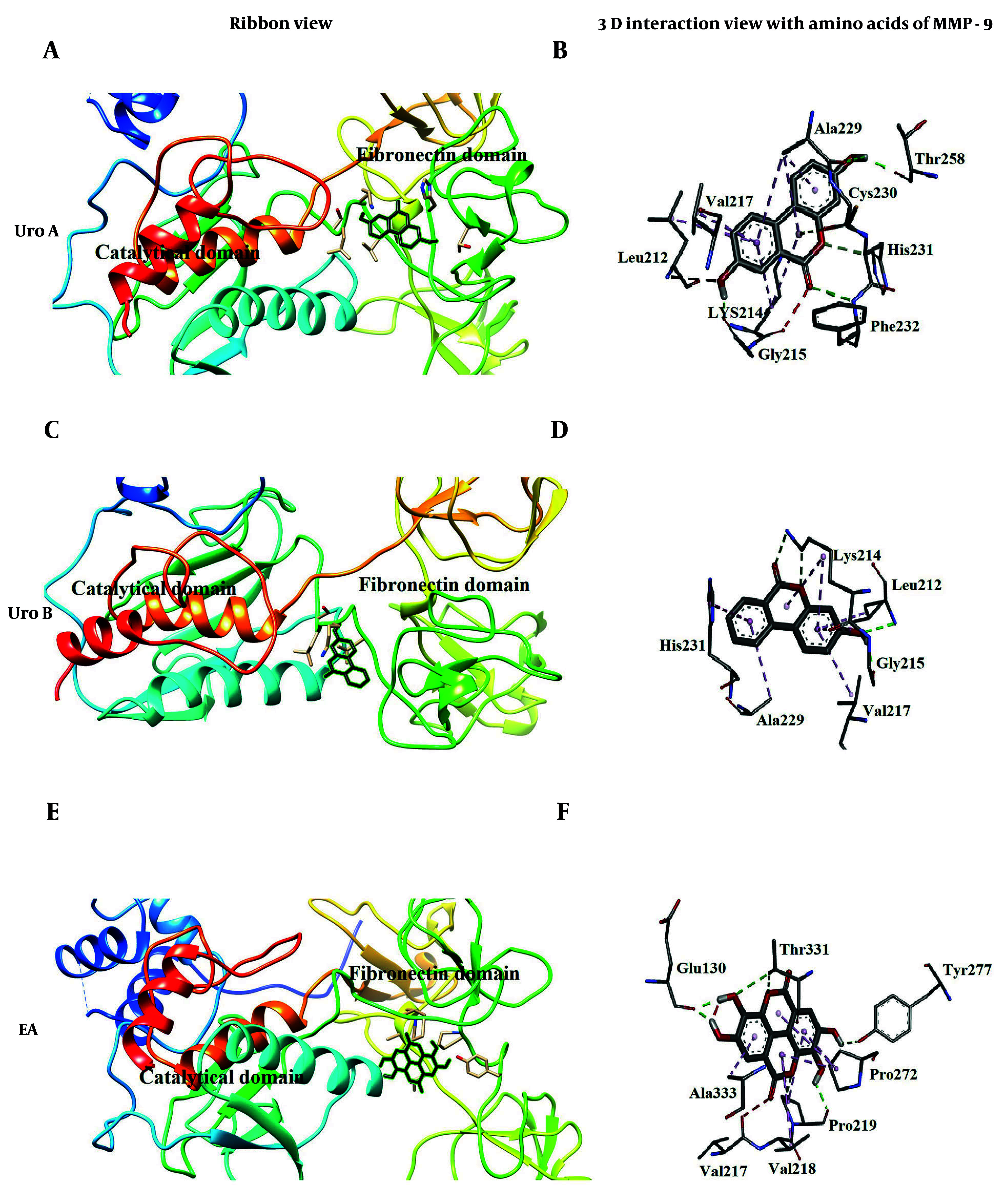
Molecular docking (35) poses [A, for urolithin A (Uro A); C, for urolithin B (Uro B); E, for ellagic acid (EA)]; and 3D interaction (B for Uro A; D for Uro B; F for EA) of ellagitannins with fibronectin domain of matrix metalloproteinase 9 (MMP-9)

Molecular docking analysis confirmed that the three inhibitor molecules could bind to the fibronectin domain as an allosteric site, with EA showing more affinity to this domain compared to Uro A and Uro B, which could tightly bind to the catalytic site. Urolithin A (Figure 7A) and Uro B (Figure 7C), with binding energies of -7.44 kcal/mol and -6.72 kcal/mol, respectively, interacted with the loop connecting the βV sheet of the catalytic and the first repeat of fibronectin domains. Both Uro A (Figure 7B) and Uro B (Figure 7D) occupied approximately the same position (Gly215, Leu212, Lys214, Val217, His231, and Ala229), except for some additional interactions formed between Uro A and the receptor protein (MMP-9). The EA (Figure 7E and F) bound to the cavity formed between the catalytic and fibronectin domains, making connections with amino acids of both domains at a different allosteric site separate from the active site compared to urolithins, with a calculated binding energy of -7.35 kcal/mol.

**Figure 8.**
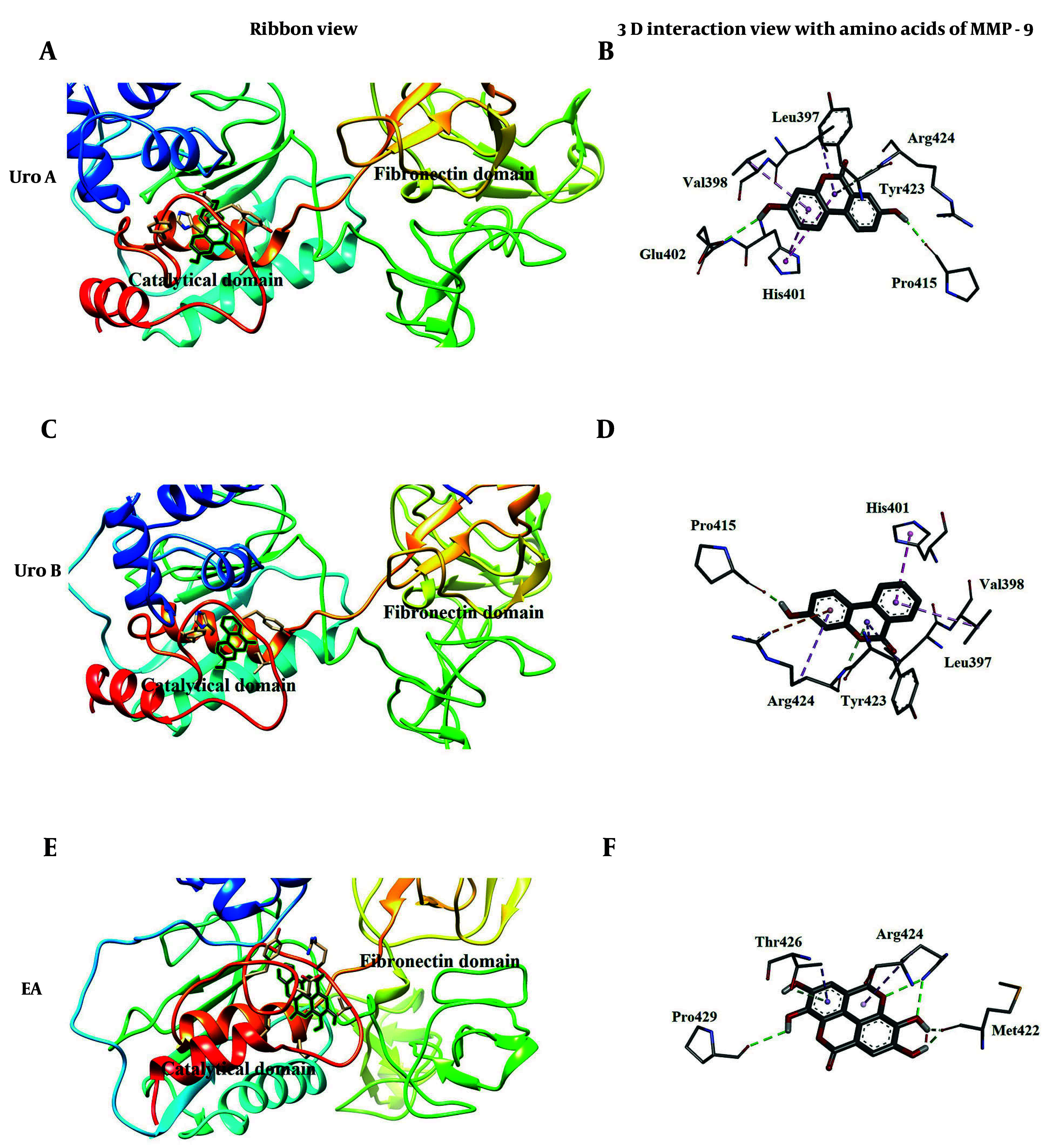
Molecular docking poses [A for urolithin A (Uro A); C for urolithin B (Uro B); E for ellagic acid (EA)] and 3D interaction (B for Uro A; D for Uro B; F for EA) of ellagitannins with catalytic domain of matrix metalloproteinase 9 (MMP-9)

The second docking position for ellagitannins, tested in this research, confirmed that they bound to the catalytic domain of the enzyme. Uro A (Figure 8A) and Uro B (Figure 8C), with binding energies of -8.41 kcal/mol and -8.54 kcal/mol, respectively, and EA (Figure 8E), with a binding energy of -6.19 kcal/mol, occupied an approximately similar binding position at the catalytic domain of the enzyme. Uro A, Uro B, and EA were fitted into the S'1 pocket of the active site, but EA had a more outward position compared to urolithins. Uro A (Figure 8B) and Uro B (Figure 8D) had similar interaction points except for one additional hydrogen bond for Uro A with residue Glu402, which plays a significant role in the catalytic mechanism of MMP-9. Matrix metalloproteinase 9 (MMP-9) enzyme amino acid residues interacting with the three ligands at the catalytic domain of the enzyme are shown in Table 2. The more outward position of EA in the S'1 pocket compared to the binding positions of Uro A and Uro B led to its interaction with different amino acid residues, except Arg 424 (Figure 8F). This outward position of EA can likely be explained by its more rigid and bulkier structure compared to urolithins, as well as its more hydrophilic structure compared to urolithins, which is less compatible with the hydrophobic nature of the S'1 pocket of the catalytic domain.

## 5. Discussion

The anticancer effects of EA and urolithins are primarily attributed to their ability to inhibit cancer cell proliferation. A wide variety of cancer cell lines in vitro have been studied in this context. Urolithins inhibit cell proliferation via cell-cycle blockage and induction of apoptosis (28). To our knowledge, the effects of these ellagitannins on the activity of MMP-9, whose elevated expression is commonly observed in invasive and tumorigenic cancers, are poorly examined.

In this research, the activity of the rhMMP-9 enzyme as a protease in the presence and absence of three natural compounds (EA, Uro A, and Uro B) revealed that all compounds inhibit rMMP-9 activity, with Uro B demonstrating the strongest inhibition potential. Determined IC_50_ values showed that Uro B has the lowest IC_50_ (13.17 µM), with this value increasing from EA (17.14 µM) to Uro A (33.29 µM). All three compounds demonstrated a mixed type of inhibition.

The binding affinity of EA, Uro A, and Uro B against rhMMP-9 was confirmed by SPR and molecular docking studies conducted in this research. KD values found for Uro A-MMP-9, Uro B-MMP-9, and EA-MMP-9 complexes revealed a stronger affinity of Uro B to the rMMP-9 enzyme compared to the other natural compounds investigated in this research.

As far as we know, experimental research on the inhibition of the catalytic function of the MMP-9 enzyme is limited, and in silico docking studies of EA and urolithins with crystallized MMP-9 are not well explored. Recent computational studies showed good binding potential of other natural herbal compounds, such as sappanol and sventenin, with the S_1_^1^ pocket of crystallized MMP-9 (29). In silico screening among natural compounds revealed four inhibitors interacting with the active site S_1_^1^ pocket of the enzyme, targeting the Zn atom of the active site and entering the S_1_^1^ pocket with their hydrophobic long-chain group. One of these inhibitors (NP-013380) showed an IC_50_ of 26.94 µM (30). Four natural coumarin inhibitors were reported to bind crystallized MMP-9 with binding energies of -7.8, -7.3, -7.6, and -7.5 kcal/mol through hydrogen and hydrophobic interactions (31). Derivatives of cinnamic acid (cynarin, chlorogenic acid, and rosmarinic acid) were found as potential inhibitors of MMP-9, binding to the catalytic domain of the enzyme and demonstrating binding energies less than -10 kcal/mol (32).

Our molecular docking study also showed direct interaction of Uro A and Uro B with the S'1 pocket of the active site in the catalytic domain. However, EA occupied a more outward position relative to the S_1_^1^ pocket, which can be explained by its more rigid and bulky structure compared to Uro A and Uro B, and its more hydrophilic structure that is less compatible with the hydrophobic nature of the S_1_^1^ pocket. Additionally, EA showed a higher binding energy value (-6.19 kcal/mol) and less affinity to this position compared to Uro A (-8.41 kcal/mol) and Uro B (-8.54 kcal/mol).

The second preferable binding position of ellagitannins primarily involves allosteric sites located on the loop connecting the fibronectin and catalytic domains of MMP-9 for Uro A and Uro B, and the cavity formed between the catalytic and fibronectin domains for EA. The fibronectin domain in gelatinases is responsible for recognition and gelatin binding (33, 34). This fibronectin domain in gelatinases (MMP-9 and MMP-2) contains important exosites, accountable for the degradation of some substrates. These exosites are considered new binding sites, and efforts are being made to design inhibitors that can bind to these exosites without interfering with catalytic Zn^2+^ (34). Both Uro A and Uro B occupied approximately the same place on the fibronectin domain, being only located at an angle with respect to each other. EA showed good prevalence to this domain with a binding energy of -7.35 kcal/mol compared to Uro A (-7.44 kcal/mol) and Uro B (-6.72 kcal/mol).

Whether these binding positions at the fibronectin domain for EA, Uro A, and Uro B correspond to the aforementioned exosites needs further investigation. Comparing the kinetic parameters and results for molecular docking of Uro A and Uro B revealed that the additional hydroxyl group in Uro A causes positive binding energy, which refers to a loss of binding to the enzyme, possibly due to steric repulsion. This reduced affinity also causes a decrease in inhibition efficiency (higher IC_50_ value).

Considering the results of binding to both investigated positions, we could conclude that inhibitors binding to the catalytic site are more efficient because Uro A and Uro B, which bind to the catalytic site, could inhibit the enzyme completely, whereas EA, which mainly binds to the fibronectin domain, could not inhibit protease activity completely. Conversely, EA, with its more rigid and plate-like structure, could bind to the free enzyme more tightly, but Uro A and Uro B, with their delicate and flexible structures, could bind to both the free enzyme and the ES complex. Considering the vital function of MMP-9 inside cells, it seems that partial inhibition of the enzyme by EA is more beneficial than chemicals that inhibit the enzyme completely.

## Data Availability

The dataset presented in the study is available on request from the corresponding author during submission or after its publication.
